# Characterisation of Phosphate Accumulating Organisms and Techniques for Polyphosphate Detection: A Review

**DOI:** 10.3390/s16060797

**Published:** 2016-05-31

**Authors:** Cédric Tarayre, Huu-Thanh Nguyen, Alison Brognaux, Anissa Delepierre, Lies De Clercq, Raphaëlle Charlier, Evi Michels, Erik Meers, Frank Delvigne

**Affiliations:** 1 Natural Products and Industrial Biochemistry Research Group (NPIB), Faculty of Applied Sciences, Ton Duc Thang University, 19 Nguyen Huu Tho, Tan Phong Ward, District 7, 700000 Ho Chi Minh City, Vietnam; nguyenhuuthanh@tdt.edu.vn; 2 Microbial Processes and Interactions, Bât. G1 Bio-Industries, Passage des Déportés 2, Gembloux Agro-Bio Tech, University of Liège, 5030 Gembloux, Belgium; cedric.tarayre@ulg.ac.be (C.T.); alison.brognaux@ulg.ac.be (A.B.); anissa.delepierre@ulg.ac.be (A.D.); raphaelle@charlier.net (R.C.); 3 Department of Applied Analytical and Physical Chemistry, Laboratory of Analytical Chemistry and Applied Ecochemistry, Ghent University, Coupure Links 653, B-9000 Ghent, Belgium; liesdeclercq@hotmail.com (L.D.C.); Evi.Michels@UGent.be (E.M.); Erik.Meers@UGent.be (E.M.)

**Keywords:** PAO, polyphosphate, detection, single cell technologies

## Abstract

Phosphate minerals have long been used for the production of phosphorus-based chemicals used in many economic sectors. However, these resources are not renewable and the natural phosphate stocks are decreasing. In this context, the research of new phosphate sources has become necessary. Many types of wastes contain non-negligible phosphate concentrations, such as wastewater. In wastewater treatment plants, phosphorus is eliminated by physicochemical and/or biological techniques. In this latter case, a specific microbiota, phosphate accumulating organisms (PAOs), accumulates phosphate as polyphosphate. This molecule can be considered as an alternative phosphate source, and is directly extracted from wastewater generated by human activities. This review focuses on the techniques which can be applied to enrich and try to isolate these PAOs, and to detect the presence of polyphosphate in microbial cells.

## 1. Introduction

Phosphorus is an essential element required for food production. It has been estimated that about 19.7 MT of phosphorus were extracted from phosphate rocks in 2000. About 15.3 MT were used to produce fertilizers out of this total production. The global growing population induces a progressive increase of fertilizers’ requirement, which means an increasing need for phosphate (P). In this context, the establishment of circular economy applicable to P resources appears obvious [1]. Europe does not have significant P mines and must import ores from the other continents. The total P-reserves have been estimated at 67,000 Tg, and Morocco owns 75% of these known deposits. The remaining percentage mainly belongs to China and the USA. This geopolitical context can generate unexpected increases of fertilizers’ prices, which occurred in 2008 [2]. It is, therefore, necessary to find new P sources. Some wastes contain recoverable P, such as wastewater. A growing attention has been paid to the research of new techniques to remove P from it. More specifically, enhanced biological phosphorus removal (EBPR) is currently applied in wastewater treatment plants (WWTPs). Sewage sludge resulting from wastewater treatment contains significant quantities of P. This is due to the presence of phosphate accumulating organisms (PAOs) [3]. PAOs have been studied in many research papers. Under specific conditions, these microorganisms can store P as polyphosphate (Poly-P). This ability has been found in bacteria, microalgae, and fungi [3,4,5].

This article focuses on bacterial PAOs and investigates the difficulties encountered in isolation attempts. It presents an inventory of analytical techniques which can be used to detect the presence of PAOs and Poly-P.

## 2. Techniques for the Isolation of PAOs

The methods used to detect and identify the PAOs are mainly based on the detection of Poly-P and polyhydroxyalkanoate (PHA). However, the detection of Poly-P itself is much more reliable. Prior to the detection of the polymers which are specific of PAOs (mainly Poly-P), an enrichment step of microorganisms is required. The original samples are mostly collected in WWTPs, in which the metabolism of PAOs is used to remove free phosphate from wastewater by accumulating it as Poly-P [6]. However, it is important to note that the maximal efficiency of PAOs is far from being reached in common EBPR plants.

### 2.1. Isolation of PAOs

Poly-P is the main form of phosphate accumulation. However, other forms of phosphate storage can be encountered. These components were investigated in a previous review [7]. The P storage compounds can be mineral or organic and depend on the type of microorganism. Low-solubility orthophosphate compounds are the simplest forms of P-accumulation. Some archaea can precipitate MgPO_4_OH·4H_2_O (such as *Halobacterium salinarium* and *Halorubrum distributum*). These strains concentrate P from aqueous solution during their growth phase. *Brevibacteria* can also accumulate P as intracellular NH_4_MgPO_4_·6H_2_O. Finally, cyanobacteria accumulate P in their sheaths, combined with calcium. The second type of P storage compounds is organic. Teichoic acid is synthesized by bacteria and is composed of polyol and glycosyl polyol residues bound by phosphodiester bonds. It has been suggested that this molecule is a phosphate reserve. The yeast *Kuraishia capsulata* can also store phosphate in extracellular phosphomannan [7].

However, in most microorganisms, inorganic Poly-P is the main P reserve, composed of three to several hundred phosphate units. This molecule is a polymeric reserve which also plays a role in the regulation of enzymatic activities, the expression of many genes and the stress adaptation process [7]. Many studies report trials of isolation of PAOs. *Acinetobacter* was isolated from an EBPR plant, but subsequent studies using culture-independent techniques revealed that this bacterium had a small, but significant, effect in the process. *Microlunatus phosphovorus* was also isolated and proposed as a PAO, but did not show their characteristics (presence of PHA granules). Molecular techniques allowed to identify Candidatus *Accumulibacter phosphatis* (*Rhodocyclus*-related bacteria) as an important subclass involved in P removal at the lab scale, but it was never isolated in pure culture. This subclass is considered as the model showing all the characteristics of a typical bacterial PAO. It is important to note the diversity of this subclass, including two types (I and II), and seven clades in Type II (IIA, IIB, IIC, IID, IIE, IIF and IIG). A recent paper reported the existence of two new clades (IIH and III) and concluded that the configuration of WWTPs has a direct impact on the structure of PAO communities [8]. However, the attempts of isolation of true PAOs have not been successful so far [9].

Some screening techniques have been proposed. Morohoshi *et al.* [10] developed a method based on the detection of PAOs by the apparition of a blue color due to the hydrolysis of 5-bromo-4-chloro-3-indolylphosphate on agar plates. Chaudhry and Nautiyal [11] also developed a visual technique based on the use of Toluidine blue-O dye in agar plates containing sodium citrate as the main carbon source. It is important to note that it has not been possible to isolate pure microorganisms seen as true PAOs so far, although many efforts have been made in this respect. This is due to the symbioses in microbial consortia, broken by the isolation techniques.

The isolation attempts have tested many enrichment media containing acetate and citrate as carbon sources [6,12,13,14]. Next, phosphate uptake assays must be achieved in order to confirm the P-accumulation ability of the strain isolated. At this level, the mechanisms of P-accumulation as Poly-P (aerobic /anoxic conditions) and P-release (anaerobic conditions) in bacteria have been described well. However, it must be kept in mind that many parameters affect the capacity of P-accumulation, such as time of anaerobic and aerobic/anoxic phases, pH, temperature, volatile fatty acid composition, PO_4_^3−^, SO_4_^2−^, NH_4_^+^, K^+^, Na^+^, Mg^2+^, Ca^2+^, and other metal ion concentrations [11,12,15].

These mechanisms have a direct impact on isolation strategies. Firstly, an anaerobic step is necessary to remove Poly-P before undertaking phosphate uptake assays [6]. Secondly, the detection of Poly-P can be performed only after having grown the bacterial strains in aerobic conditions.

**Figure 1:**
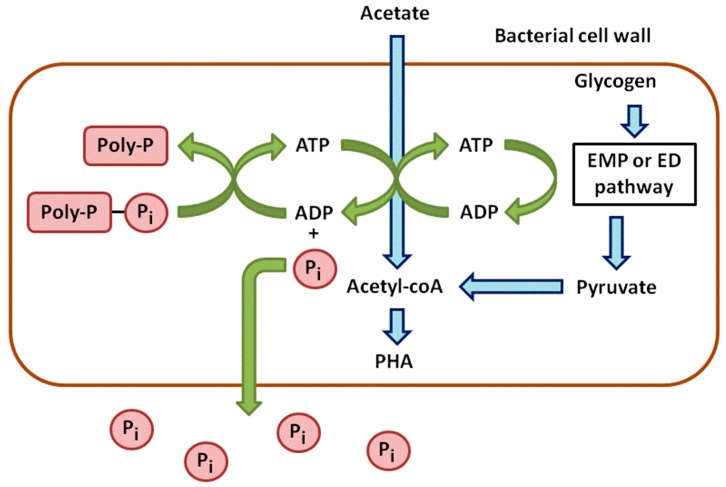
Anaerobic metabolism of phosphorus in phosphate accumulating bacteria.

Anaerobic phases can be considered as stress conditions for PAOs. They take up the carbon sources and store them to cope with a potentially long-term absence of oxygen (Figure 1). In wastewater, acetate is the main carbon source and is converted into Acetyl-CoA. This reaction requires energy provided by the hydrolysis of ATP, leading to ADP and the release of P in the medium. Acetyl-CoA is next metabolized into β-hydroxyalkanoates (PHAs), which are carbon storage compounds [9].

**Figure 2:**
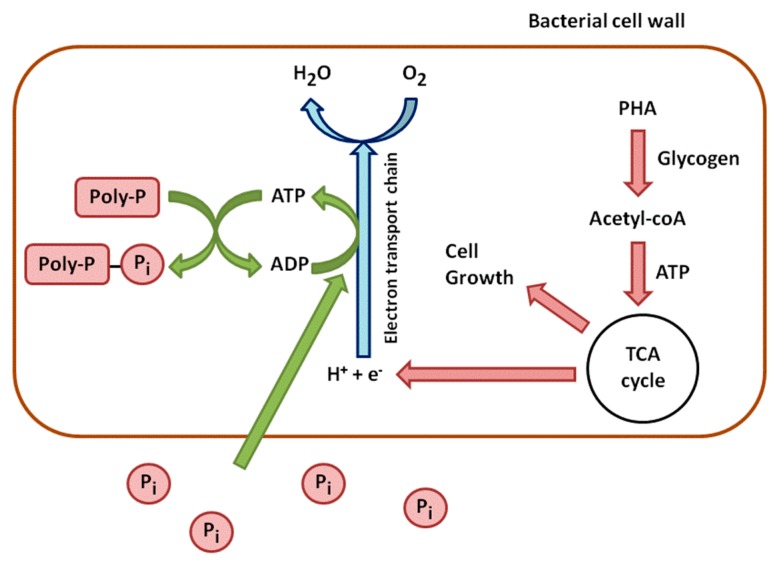
Aerobic metabolism of phosphorus in phosphate accumulating bacteria.

In aerobic conditions, this trend is reversed. PHAs are used for cell growth and the reconstitution of the Poly-P reserves (Figure 2). They are degraded, which leads to the synthesis of Acetyl-CoA, processed through the TCA cycle. The TCA cycle produces energy from oxidation and carbon for new cell growth. A part of this energy is used to take up soluble P from the environment and to incorporate it into Poly-P. Another part of carbon and energy is used to regenerate glycogen [16].

The case of phosphate accumulating microalgae and mycetes has much less been investigated. However, it has been determined that microalgae accumulate P under stress conditions in specific organites: the acidocalcisomes [17].

The techniques applied to detect Poly-P in microorganisms are diversified and were reviewed in several articles [18,19,20,21,22,23]. Here, we propose a global overview of the methods which can be applied to the detection of PAOs and Poly-P.

### 2.2. Poly-P Detection and Identification of PAOs

The methods used to detect Poly-P in microbial cells are diversified. The technique used depends on the information required (detection, quantification, degree of polymerization (DP)). They can focus on the PAOs or the Poly-P molecule itself. The methods applicable are summarized in Table 1.

#### 2.2.1. Staining Techniques—Light and Epifluorescence Microscopy (LEM)

The most common techniques consist in dying Poly-P with chemicals and observing the Poly-P granules with a light or fluorescence microscope. These qualitative methods were the first used and are still being applied, owing to their simplicity. Loeffler’s methylene blue staining is based on the affinity of anionic Poly-P with methylene blue. The bonds between the dying molecules and Poly-P lead to a specific pink-violet color. Toluidine blue can be used instead of methylene blue because its metachromatic properties are identical. Neisser staining, the second common method, also uses methylene blue combined with a counterstaining solution (Bismark brown or Chrysoidin Y). This protocol improves the detection efficiency by increasing the contrast between Poly-P and the other cell components. Neisser staining protocol leads to a purple color for Poly-P, while the other cellular components are yellowish-brown. Regardless of what technique is used, methylene blue requires an acidic pH to improve the linkages with Poly-P [19]. Neutral red is a pH-sensitive dye, as well. It is used as a probe which detects acidic cellular compartments, such as the ones which contain Poly-P (under the form of “volutin” granules). The neutral form of neutral red is membrane-permeable, while the protonated form is not. Consequently, the acidic compartments tend to accumulate neutral red and can be observed through fluorescence microscopy. The accumulation can be detected on the basis of the absorbance shift: neutral red shows a λ_max_ of 450 nm at pH 8.1 while its protonated form shows a λ_max_ of 535 nm at pH 5.3 [24]. The case of 4′,6-diamidino-2-phenylindole (DAPI), another dying molecule, has been reported in many research articles. This dye stains Poly-P granules with a yellow fluorescence when used at high concentrations (see the part “Flow cytometry”). However, DAPI is not specific: it also stains other polymeric ions, such as DNA and lipids [20]. DAPI-DNA fluorescence is blue, but both DAPI-Poly-P and DAPI-lipids show a yellow fluorescence. The fluorescence of DAPI-lipids is shorter in time than the one of DAPI-Poly-P [25]. High DAPI concentrations induce a high fluorescence background, and lower DAPI concentrations coupled with a longer incubation time can be applied [19]. Due to its lack of specificity, the detection of Poly-P with DAPI can be performed after having checked that the reserves of PHA have been totally consumed. This storage polymer can be detected with Sudan black and Nile blue [19]. Other marginal dyes were mentioned in some articles, such as tetracycline (fluorescence based on the linkage with Poly-P) and Fura-2 (fluorescence depending on the competition of Mn^2+^ affinity with Fura-2 and Poly-P) [26,27].

#### 2.2.2. Flow Cytometry (FC)

Flow cytometry has been cited in many research articles relating to the analysis of Poly-P, mainly with DAPI. The staining reaction is exactly the same as in fluorescence microscopy, but flow cytometry brings much more data [28]. DAPI stains the cells with Poly-P contents higher than 400 μmol/g (dry weight) under a concentration of at least 18 μM (5 to 50 μg/mL). It is considered that a lower concentration in DAPI (0.24 to 5 μM) leads to a blue fluorescence related to bacterial DNA only, while the fluorescence due to Poly-P is yellow and induced by higher DAPI concentrations [27]. Flow cytometry allows the measuring and analysis of multiple physical characteristics, and the particles of interest can be separated from another to make further analyses through cell sorting [28]. Flow cytometry has already been used to detect Poly-P in bacterial cells but also in mammal cells [29]. This technique can be used to locate the Poly-P granules inside the living cells. Many research articles cited DAPI in the topic of Poly-P research [27,28,29,30,31]. Tetracycline can also be used to detect Poly-P through flow cytometry. This antibiotic and its derivatives are usually used in medicine as a labelling molecule for the assessment of calcium deposition. The association of diamagnetic divalent cations (Mg^2+^, Ca^2+^) with tetracycline leads to a green fluorescence (emission wavelength of 515 nm), with an excitation wavelength of 390 nm at a neutral pH [27]. This protocol was applied in several research articles [27,32,33].

#### 2.2.3. Fluorescence *in Situ* Hybridization (FISH) Analysis

Fluorescence *in situ* hybridization (FISH) is rather linked to the microorganisms themselves and is based on the use of labelled probes which can detect DNA or RNA molecules *in situ*. This technique is applicable to the identification of microorganisms, the study of gene expression and the study of ecophysiology, which is particularly interesting in the case of PAOs [34]. However, the technique suffers from disadvantages due to its specificity: the sequence of the target molecules and their abundance are required, and the probes must also penetrate the cells with a sufficient efficiency [28]. Albertsen *et al.* [35] used quantitative FISH with 32 oligonuclotide probes to investigate the composition of the bacterial communities in Danish EBPR plants. FISH analysis allows targeting of specific bacterial clades. Such probes were used to detect the presence of *Accumulibacter*, a well-known PAO [36,37]. It can also be used to determine the location of PAOs in complex samples [38]. FISH analysis may be combined with other techniques such as in the study of Liu *et al.* [39], which used DAPI staining, electron microscopy, and X-ray analysis as complementary techniques. The labelled cells can be observed through microscopy techniques [40], but flow cytometry is also applicable. This combination was used in the study of Kim *et al.* [41] to separate four *Accumulibacter* clades with Fluorescence-Activated Cell Sorting (FACS). Another possible combination is FISH/micro-autoradiography ((MAR)-FISH), which used labelled substrates (^3^H, ^14^C) to investigate the assimilation of nutrient sources according to the conditions used [42].

#### 2.2.4. Extraction Procedures and Polyphosphate Quantification (EXT)

Many articles report extraction procedures of Poly-P followed by its quantification. This procedure is applied to microorganisms the ability to accumulate Poly-P of which has been confirmed. Several problems must be considered: inorganic Poly-P is usually combined with other macromolecules, such as nucleic acids and proteins; the Poly-P solubility depends on the chain length and the presence of cations; the cellular breakdown required to extract Poly-P may lead to undesired modifications, such as changes in chain length, concentrations, and linkage to inorganic molecules. The extraction procedures were reviewed before [20]. The extraction of Poly-P from living cells and the subsequent phosphate (resulting from Poly-P hydrolysis) measurement can be made through different techniques. An extraction by NaOH followed by an acidic hydrolysis with HCl was performed by Aravind *et al.* [12], before phosphate measurement by the method of molybdate reagent and ascorbic acid. Another research article reported the extraction of different Poly-P fractions (acid-soluble with HClO_4_, salt-soluble with NaClO_4_ and HClO_4_, and alkali-soluble with NaOH) from *Saccharomyces cerevisiae*, before the digestion by HClO_4_ [43,44]. Poly-P can also be extracted by the method of alkaline hypochlorite [11]. Jimenez-Nuñez *et al.* [29] used a digestion technique using RNase and DNase followed by an extraction with phenol-chloroform. Next, exopolyphosphatase was used to assess the Poly-P concentration. The extraction with boiling water was also tested, but this technique leads to a partial hydrolysis of Poly-P, which is not desired until the true hydrolysis step [20]. The analysis of phosphate resulting from the hydrolysis step can also be achieved by ion chromatography and inductively coupled plasma (ICP) atomic emission spectrometry (AES) [22,23].

#### 2.2.5. Polyacrylamide Gel Electrophoresis (PAGE)

Gel electrophoresis can provide data about the DP of Poly-P and requires a Poly-P extraction procedure. The samples must be treated to obtain a soluble form of Poly-P. Cation-exchange resins can be used to reach these conditions, and the migration of standards allows to assess the size of Poly-P molecules. A 6%–30% polyacrylamide gel with 7 M urea was used in several research articles [29,43,44]. Next, the gels can be stained for visualization (Toluidine blue) [44]. Another publication reports the use of PAGE added with DAPI, which led to a yellow fluorescence, while nucleic acids and glycosaminoglycans fluoresced blue [45].

#### 2.2.6. Electron Microscopy (EM)

Electron microscopy is a powerful technique which allows to visualize and locate the Poly-P granules inside cells. The technique used alone cannot quantify the concentrations in Poly-P, but confirms the presence of Poly-P granules. Scanning electron microscopy (SEM) is usually used with other techniques, such as staining protocols [22,23]. Scanning transmission electron microscopy (STEM) can be used to detect high-density granules, such as Poly-P [46]. Electron microscopes are usually equipped with X-ray detectors, which can confirm the elementary composition of the material and the composition of Poly-P granules [43]. Another article reports a complete analysis of phosphate granules in fossil bacteria through several microscopy techniques (SEM, STEM, and Scanning Transmission X-ray Microscopy) [47]. Such a combination was also used in another work which studied the accumulation of Poly-P in *Comamonadaceae* through SEM combined with energy dispersive X-ray spectroscopy [48]. A metallization can be applied to the samples to improve the performance of electron microscopy, with osmium tetroxide as in a previous study [13]. Another article investigated the structure of precipitated phosphate minerals (struvite) produced by phosphate forming bacteria through SEM and field-emission scanning electron microscopy (FESEM) [49].

#### 2.2.7. X-Ray Analysis Techniques (X-RAY)

X-ray analysis provides information about the composition of the samples and is usually combined with electron microscopes. Energy-dispersive X-ray microanalysis was previously used to assess the composition of Poly-P granules observed through electron microscopy (abbreviated by EM-EDX) [46]. Such combination was used in many studies [43,47,48]. However, the treatments applied before the observation stage must be limited to prevent P losses from the cells [20]. X-ray analyses highlighted the effect of redox conditions on different types of Poly-P: the polymers associated with Mg and K are more easily metabolized than the ones associated with Ca, which means that this latter type of phosphate storage is not influenced by the presence or absence of aerobic conditions [20]. Another useful combined technique is X-ray fluorescence spectromicroscopy, a combination of X-ray spectroscopy and X-ray microscopy. This method was found to be able to elucidate the spatial concentration and speciation of elements at submicron scale in minimally-prepared particulate samples including individual cells. It can analyze thicker samples than electron microscopy and is less restrictive during the preparation procedure [22,23]. The technique developed by Rivadeneyra *et al.* [49] used an X-ray diffractometer to study struvite crystals produced in culture media. This device allowed to determine the qualitative and quantitative composition of the salts produced by bacteria [49].

#### 2.2.8. Nuclear Magnetic Resonance Spectroscopy (NMRS)

This non-invasive and non-destructive technique requires labelled substrates (^31^P) and is applied to PAOs to study their physiology. The labelled substrates are included in Poly-P granules, which allows assessing the substrate degradation, and the synthesis of end-products and metabolites in only one sample [28]. The method can be applied to liquid (culture media) and solid samples and provides an excellent overview of the molecules containing phosphorus (Poly-P but also orthophosphate, pyrophosphate, phosphate mono- and di-esters, phosphonates). However, the interference caused by the heterogeneous physical and chemical properties of the samples and the association of P containing molecules with metallic ions (iron, manganese) must be highlighted. Another point is the similarity of linkage in Poly-P and other molecules containing phosphoanhydride bonds, such as nucleotides. Consequently, NMR usually provides results of the distribution of chemical linkages detected in the sample considered without providing data about the real Poly-P concentration [23]. NMR can also be coupled with bioreactors to follow up the concentrations in P containing metabolites [21]. A recent research article investigated the P transfers under aerobic and anaerobic conditions through NMR spectroscopy and studied the extracellular polymeric substances, focusing on Poly-P [50]. This work showed that NMR can be used as a relevant quantitative technique applicable to the study of PAOs.

#### 2.2.9. RAMAN Spectromicroscopy (RAM)

RAMAN spectromicroscopy requires a simple preparation protocol of samples and may be used with complementary molecular techniques [22,23]. Near-infrared reflectance spectroscopy was first found to be able to provide precious data about carbon, carbonate, N, and P in sediments [51]. RAMAN spectromicroscopy is a related technique which allowed to identify and quantify Poly-P and other storage polymers involved in phosphate accumulation in individual bacterial cells. It also identified orthophosphate, trimers, hexamers, and other oligomers on the basis of the specificity of spectra [23]. RAMAN spectroscopy was also found to be a reliable technique applicable to the study of microbiota composition [52]. More specifically, a research article reports the successful use of RAMAN spectromicroscopy to investigate the dynamics of intracellular Poly-P in PAOs in EBPR process [53]. Further research was applied to Poly-P, PHA, and glycogen, and developed a reliable technique to assess the intracellular distribution and dynamics of these polymers in different metabolic stages of the EBPR process [54]. Another research paper described RAMAN spectromicroscopy as a powerful tool to identify bacteria as PAOs or GAOs on the basis of their intracellular polymers [23]. Indeed, GAOs and PAOs are known to compete to consume carbon sources, and their metabolic properties relating to phosphorus are completely different. GAOs do not participate in the P-removal process [15].

#### 2.2.10. Enzyme Assays (EA)

The metabolism of Poly-P depends on the activity of different types of enzymes. It is synthesized in bacterial cells by Poly-P kinases (PPK1 and PPK2) and degraded by exopolyphosphatase (PPX). A first technique to detect Poly-P in samples consists in using a yeast exopolyphosphatase on the extract and measuring the resulting phosphate concentration. Another enzyme assay is based on the action of PPK, which converts ADP to ATP in the presence of Poly-P [21]. After the enzymatic hydrolysis, the reaction products can be analyzed by thin-layer chromatography or gel electrophoresis [46]. Other techniques based on the activity of Poly-P-glucokinase and Poly-P AMP phosphotransferase were also reported [20]. Such enzyme assays require a good quality of the extract supposed to contain Poly-P.

#### 2.2.11. Cryoelectron Tomography and Spectroscopic Imaging (CTSI)

This technique was used in the study of Comolli *et al.* [55] to investigate polymeric structures in *Caulobacter crescentus* and *Deinococcus grandis*. Here, the study of subcellular structure and subcellular bodies in whole bacteria was undertaken. This combination led to the identification of P-rich and carbon-rich bodies in *Caulobacter crescentus*, and provided structural information without altering the granules. P-rich granules were also detected in *Deinococcus grandis*.

#### 2.2.12. Mass Spectrometry (MS)

Electrospray ionization mass spectrometry (ESI-MS) was tested on orthophosphate, pyrophosphate, tripolyphosphate, and tetrapolyphosphate [21]. This technique shows a high selectivity and does not require pre-separation steps. However, this method was not really investigated on samples extracted from EBPR experiments or pure cultures of PAOs.

#### 2.2.13. Proteic Affinity (PA)

Saito *et al.* [56] developed a proteic affinity technique which leads to the detection of Poly-P. The principle is based on the use of a recombinant Poly-P binding domain (PPBD) of an exopolyphosphatase from *Escherichia coli*. The PPBD showed a high affinity for long-chain Poly-P and a low affinity for short-chain Poly-P. The technique was found to be able to locate Poly-P by an immunocytochemical method.

#### 2.2.14. “Omics” Techniques (OMICS)

Omics techniques have been widely used in many studies over the past decade. Here, we present some applications of those data-rich methods to the study of PAOs. Metagenomics is mainly based on the analysis of 16S rRNA extracted from complex microbiotas, and is perfectly applicable to the study of the strains involved in the EBPR process [57]. This technique was combined with quantitative FISH in a previous work and was applied to the bacterial community involved in EBPR process [35]. Metatranscriptomics, based on the analysis of mRNA sequences, has also been used to investigate the EBPR process. Such study was successfully applied to the research of Poly-P kinase sequences and focused on *Accumulibacter* [58]. Finally, metaproteomics provides data about the expression of proteins in complex samples, but the results obtained by this method must be considered carefully. Genes are regulated at the transcriptional, translational, and post-translational levels, and another point comes from the short half-life of gene products. Consequently, metaproteomics’ data should not be considered alone [57]. The study of Wilmes *et al.* [59] reported a metaproteomic analysis of bacterial consortia according to the efficiency of the EBPR process. A successful EBPR process led to the detection of a majority of *Accumulibacter*, while the failure of the process led to a majority of *α-Proteobacteria* [58,59]. The study of Wexler *et al.* [60] used radiolabelled metaproteomics to investigate the effect of anaerobic and aerobic conditions on the EBPR process. Another combined study was based on proteogenomics and assessed the role of *Accumulibacter* in the EBPR process, taking the other co-existing strains into account. The metaproteome was found to be very close in both aerobic and anaerobic conditions, and denitrification, fatty acid cycling and the glyoxylate bypass were revealed to have an important role in the EBPR process [59].

Table 2 summarizes the specificities of each technique described above.

## 3. Conclusions

Analytical techniques focusing on the isolation or the detection of Poly-P are quite diversified. However, there is evidence that the culture of pure PAOs requires more investigation, and the enrichment of phosphate-accumulating consortia is an alternative to this constraint. This method requires a perfect understanding of the effect of environmental conditions on PAOs in order to establish phosphate accumulation-oriented consortia. This field is still lacking knowledge, although the general metabolism has been described in many publications. Flow cytometry is also a powerful technique applicable to the concentration of PAOs. Screening the genes responsible for phosphate accumulation is another solution which could lead to the engineering of phosphate-accumulating strains, more efficient than the wild-types. In these conditions, the accumulation of phosphate should be feasible without requiring specific conditions, such as aeration rate or the presence of specific components. Then, the recovery of phosphate from wastewater or other types of liquid effluents will be much easier.

## Figures and Tables

**Table 1:** Inventory of techniques applied to the detection of PAOs and Poly-P.

Technique	Information
Light and fluorescence microscopy coupled with specific staining (LEM)	Presence/absence of Poly-P
Flow cytometry (FC)	Location and quantification of Poly-P granules, cell sorting
FISH analysis (FISH)	Detection of PAOs
Extraction procedures and phosphate quantification (EXT)	Quantification of Poly-P
Polyacrylamide gel electrophoresis (PAGE)	Detection of Poly-P, determination of DP
Electron microscopy (EM)	Presence/absence of Poly-P, location and composition of Poly-P granules
X-ray analysis (X-RAY)	Composition of Poly-P granules, possible quantification
Nuclear Magnetic Resonance Spectroscopy (NMRS)	Detection of Poly-P, study of Poly-P structure
RAMAN microscopy (RAM)	Detection and quantification of Poly-P
Enzyme assays (EA)	Detection and quantification of Poly-P
Cryoelectron tomography and spectroscopic imaging (CTSI)	Detection of Poly-P, study of Poly-P structure
Mass spectrometry (MS)	Detection of Poly-P, study of Poly-P structure
Proteic affinity (PA)	Detection of Poly-P, location of Poly-P granules
“Omics techniques” (OMICS)	Study of PAOs in complex communities

**Table 2:** Specificities of techniques applied to the detection of PAOs and Poly-P.

Technique	Investment	Advantages	Disadvantages
LFM-MB	low	Simplicity, rapidity Direct visualization of Poly-P granules	Not adapted to visualize small granules Non-quantitative technique Requirement of a preparation protocol and an acidic pH Destructive technique
LFM-NR	low	Simplicity, rapidity Direct visualization of Poly-P granules	Technique targeting acidic vacuoles and not Poly-P itself Non-quantitative technique Requirement of a preparation protocol and an acidic pH Destructive technique
LFM-DAPI	low	Possibility to visualize polyhydroxyalkanoate Possibility of combination with other techniques (FC)	Expensive staining reagent Non-quantitative and destructive technique No specificity to Poly-P Importance of the noise with high DAPI concentrations
FC	high	Possibility to visualize polyhydroxyalkanoate Possibility to sort the cells in a complex sample Possibility to study the phenotypic heterogeneity Possibility to locate Poly-P inside the cells Possibility to combine different staining techniques Possibility to combine with FISH-A Possibility to adapt the technique to animal cells	Same disadvantages as LFM-DAPI Obligation to filtrate the samples Destructive technique
FISH-A	low	Possibility to combine with FC and FISH-A Possibility to study specific groups of PAOs Possibility to study gene expression Possibility to study complex microbiotas	Complexity of sample preparation Efficiency depending on the penetration of the probes inside the cells No specificity to Poly-P Destructive technique
EXT	low	Possibility to measure phosphate resulting from the hydrolysis by diversified techniques Possibility to characterize different Poly-P fractions Quantitative technique	Efficiency depending on the association of Poly-P with other molecules Effect of cations and chain length on the analysis The extraction can lead to undesired modifications of Poly-P Destructive technique
PAGE	low	Possibility to measure the size and the DP of Poly-P Possibility to combine with DAPI staining Semi-quantitative technique	The technique requires a step of Poly-P extraction Poly-P must be treated to be soluble Destructive technique
EM	high	Possibility to locate Poly-P inside the cells Possibility to combine the technique with staining protocols and X-RAY Possibility to observe Poly-P granules inferior to 100 nm	Complexity of sample preparation Non quantitative and destructive technique SEM may be not adapted to observe internal Poly-P granules
X-RAY	high	Powerful combination with EM Quantitative analysis Possibility to combine with X-ray fluorescence spectromicroscopy leading to the study of the repartition of Poly-P inside the cells	Possible loss of Poly-P during the preparation protocol Destructive technique
NMRS	high	Global assessment of phosphorus metabolism inside the cells Applicability to liquid and solid samples Possibility to use the method as a follow-up technique of bioreactors Nondestructive technique	Tagged substrates required Frequency of interferences due to the medium No specificity to Poly-P but measurement of phosphodiester linkages
RAM	high	Simplicity of preparation protocol Quantitative detection of Poly-P and other storage polymers Applicability to the study of microbial consortia Distinction of PAOs and GAOs	Weakness of RAMAN signal which requires an adapted device Interferences due to impurities and fluorescence
EA	low	Quantitative technique Possibility to assess the metabolism of Poly-P extracts	Complexity of preparation of samples to avoid an inactivation of enzymes due to impurities Complexity of enzymatic reaction, less simple than with simple chemical reagents Destructive technique
CTSI	high	Possibility to measure the diameter of Poly-P granules with a high accuracy Possibility to observe many cellular structures Nondestructive technique	Complexity of sample preparation Non quantitative and visual technique
MS	high	Possibility to characterize different Poly-P fractions High selectivity and sensitivity Simple sample preparation protocol	Destructive technique The technique requires standard samples
PA	low	Quantitative technique Possibility to locate Poly-P High resolution when the technique is combined with EM	Complexity of sample preparation The technique requires antibodies specific to Poly-P
OMICS	high	Global overview of microbial consortia Possible combination with other techniques (FISH-A) Possibility to assess the evolution of complex consortia in time and according to specific conditions	Not adapted to measure the Poly-P content Requirement of up-to-date databases

## Abbreviations

The following abbreviations are used in this manuscript:

ADP	Adenosine Diphosphate
ATP	Adenosine Triphosphate
CTSI	Cryoelectron Tomography and Spectroscopic Imaging
DAPI	4′,6-Diamidino-2-Phenylindole
DP	Degree of Polymerisation
EA	Enzyme Assays
EBPR	Enhanced Biological Phosphorus Removal
EM	Electron Microscopy
EM-EDX	Electron Microscopy—Energy-dispersive X-ray
ESI-MS	Electrospray Ionisation Mass Spectrometry
EXT	Extraction procedures
FACS	Fluorescence-Activated Cell Sorting
FC	Flow Cytometry
FESEM	Field-Emission Scanning Electron Microscopy
FISH	Fluorescence *in Situ* Hybridization
GAO	Glycogen Accumulating Organism
ICP-AES	Inductively Coupled Plasma—Atomic Emission Spectrometry
LFM	Light and Epifluorescence Microscopy
MAR	Micro-autoradiography
MS	Mass Spectrometry
MT	Megaton
NMRS	Nuclear Magnetic Resonance Spectroscopy
OMICS	“Omics” techniques
P	Phosphate
PA	Proteic Affinity
PAGE	Polyacrylamide Gel Electrophoresis
PAO	Phosphate Accumulating Organism
PHA	Polyhydroxyalkanoate
Poly-P	Polyphosphate
PPBD	Polyphosphate Binding Domain
PPK	Polyphosphate kinase
PPX	Exopolyphosphatase
RAM	Raman spectromicroscopy
SEM	Scanning Electron Microscopy
STEM	Scanning Transmission Electron Microscopy
TCA	Tricarboxylic acid
Tg	Teragram
WWTP	Wastewater Treatment Plant
X-RAY	X-ray analysis techniques

## References

[1] Wilfert P., Kumar P.S., Korving L., Witkamp G.-J., van Loosdrecht M.C.M. (2015). The relevance of phosphorus and iron chemistry to the recovery of phosphorus from wastewater: A review. Environ. Sci. Technol..

[2] Schoumans O.F., Bouraoui F., Kabbe C., Oenema O., van Dijk K.C. (2015). Phosphorus management in Europe in a changing world. Ambio.

[3] Hirota R., Kuroda A., Kato J., Ohtake H. (2010). Bacterial phosphate metabolism and its application to phosphorus recovery and industrial bioprocesses. J. Biosci. Bioeng..

[4] Powell N. (2009). Biological Phosphorus Removal by Microalgae in Waste Stabilisation Ponds.

[5] Ye Y., Gan J., Hu B. (2015). Screening of Phosphorus-Accumulating Fungi and Their Potential for Phosphorus Removal from Waste Streams. Appl. Biochem. Biotechnol..

[6] Bao L.L., Li D., Li X.K., Huang R.X., Zhang J., Lu Y., Xia G.Q. (2007). Phosphorus accumulation by bacteria isolated from a continuous-flow two-sludge system. J. Environ. Sci..

[7] Kulakovskaya T.V., Vagabov V.M., Kulaev I.S. (2012). Inorganic polyphosphate in industry, agriculture and medicine: Modern state and outlook. Process. Biochem..

[8] Mao Y., Graham D.W., Tamaki H., Zhang T. (2015). Dominant and Novel Clades of Candidatus Accumulibacter phosphatis in 18 Globally Distributed Full-Scale Wastewater Treatment Plants. Sci. Rep..

[9] Oehmen A., Lemos P.C., Carvalho G., Yuan Z., Keller J., Blackall L.L., Reis M.A.M. (2007). Advances in enhanced biological phosphorus removal: From micro to macro scale. Water Res..

[10] Morohoshi T., Yamashita T., Kato J., Ikeda T., Takiguchi N., Ohtake H., Kuroda A. (2003). A Method for Screening Polyphosphate-Accumulating Mutants Which Remove Phosphate Efficiently from Synthetic Wastewater. J. Biosci. Bioeng..

[11] Chaudhry V., Nautiyal C.S. (2011). A high throughput method and culture medium for rapid screening of phosphate accumulating microorganisms. Bioresour. Technol..

[12] Aravind J., Saranya T., Kanmani P. (2015). Optimizing the Production of Polyphosphate from Acinetobacter Towneri. Glob. J. Environ. Sci. Manag..

[13] Khoi L.Q. (2013). Isolation and Phylogenetic Analysis of Polyphosphate Accumulating Organisms in Water and Sludge of Intensive Catfish Ponds in the Mekong Delta, Vietnam. Am. J. Life Sci..

[14] Naili O., Benounis M., Benammar L. (2015). Screening of Bacteria Isolated from Activated Sludges for Phosphate Removal from Wastewater. J. Appl. Environ. Biol. Sci..

[15] Yuan Z., Pratt S., Batstone D.J. (2012). Phosphorus recovery from wastewater through microbial processes. Curr. Opin. Biotechnol..

[16] Tchobanoglous G., Stensel D., Tsuchihashi R., Burton F., Eddy M. (2013). Wastewater Engineering: Treatment and Resource Recovery.

[17] Abdulsada Z.K. (2014). Evaluation of Microalgae for Secondary and Tertiary Wastewater Treatment. Ph.D. Thesis.

[18] Eixler S., Selig U., Karsten U. (2005). Extraction and detection methods for polyphosphate storage in autotrophic planktonic organisms. Hydrobiologia.

[19] Serafim L.S., Lemos P.C., Levantesi C., Tandoi V., Santos H., Reis M.A.M. (2002). Methods for detection and visualization of intracellular polymers stored by polyphosphate-accumulating microorganisms. J. Microbiol. Methods.

[20] Hupfer M., Glöss S., Schmieder P., Grossart H.P. (2008). Methods for detection and quantification of polyphosphate and polyphosphate accumulating microorganisms in aquatic sediments. Int. Rev. Hydrobiol..

[21] Rao N.N., Gómez-García M.R., Kornberg A. (2009). Inorganic polyphosphate: Essential for growth and survival. Annu. Rev. Biochem..

[22] Majed N., Chernenko T., Diem M., Gu A.Z. (2012). Identification of functionally relevant populations in enhanced biological phosphorus removal processes based on intracellular polymers profiles and insights into the metabolic diversity and heterogeneity. Environ. Sci. Technol..

[23] Majed N., Li Y., Gu A.Z. (2012). Advances in techniques for phosphorus analysis in biological sources. Curr. Opin. Biotechnol..

[24] Ezawa T., Smith S.E., Smith F.A. (2001). Differentiation of polyphosphate metabolism between the extra- and intraradical hyphae of arbuscular mycorrhizal fungi. New Phytol..

[25] Pandolfi D., Dumas M.D. (2006). Caractérisation Morphologique et Physiologique de la Biomasse des Boues Activées par Analyse d’ Images.

[26] Lorenz B., Münkner J., Oliveira M.P., Leitão J.M., Müller W.E., Schröder H.C. (1997). A novel method for determination of inorganic polyphosphates using the fluorescent dye fura-2. Anal. Biochem..

[27] Günther S., Trutnau M., Kleinsteuber S., Hause G., Bley T., Röske I., Harms H., Müller S. (2009). Dynamics of polyphosphate-accumulating bacteria in wastewater treatment plant microbial communities detected via DAPI (4′,6′-diamidino-2- phenylindole) and tetracycline labeling. Appl. Environ. Microbiol..

[28] Günther S., Röske I., Bley T. (2011). Population Structure and Dynamics of Polyphosphate Accumulating Organisms in a Communal Wastewater Treatment Plant.

[29] Jimenez-Nuñez M.D., Moreno-Sanchez D., Hernandez-Ruiz L., Benítez-Rondán A., Ramos-Amaya A., Rodríguez-Bayona B., Medina F., Brieva J.A., Ruiz F.A. (2012). Myeloma cells contain high levels of inorganic polyphosphate which is associated with nucleolar transcription. Haematologica.

[30] Günther S., Hübschmann T., Rudolf M., Eschenhagen M., Röske I., Harms H., Müller S. (2008). Fixation procedures for flow cytometric analysis of environmental bacteria. J. Microbiol. Methods.

[31] Miyauchi R., Oki K., Aoi Y., Tsuneda S. (2007). Diversity of nitrite reductase genes in “Candidatus accumulibacter phosphatis”-dominated cultures enriched by flow-cytometric sorting. Appl. Environ. Microbiol..

[32] Günther S., Koch C., Hübschmann T., Röske I., Müller R.A., Bley T., Harms H., Müller S. (2012). Correlation of community dynamics and process parameters as a tool for the prediction of the stability of wastewater treatment. Environ. Sci. Technol..

[33] Mehlig L., Petzold M., Heder C., Gunther S., Muller S., Eschenhagen M., Roske I., Roske K. (2013). Biodiversity of Polyphosphate Accumulating Bacteria in Eight WWTPs with Different Modes of Operation. J. Environ. Eng..

[34] Chen H., Wang D., Li X., Yang Q., Luo K. (2013). Biological phosphorus removal from real wastewater in a sequencing batch reactor operated as aerobic/extended-idle regime. Biochem. Eng. J..

[35] Albertsen M., Benedicte L., Hansen S., Saunders A.M., Nielsen P.H. (2012). A metagenome of a full-scale microbial community carrying out enhanced biological phosphorus removal. ISME J..

[36] Broch S.P. (2008). Operation and Control of SBR Processes for Enhanced Biological Nutrient Removal from Wastewater.

[37] Burow L.C., Mabbett A.N., Blackall L.L. (2008). Anaerobic glyoxylate cycle activity during simultaneous utilization of glycogen and acetate in uncultured Accumulibacter enriched in enhanced biological phosphorus removal communities. ISME J..

[38] De Kreuk M.K., Heijnen J.J., van Loosdrecht M.C.M. (2005). Simultaneous COD, nitrogen, and phosphate removal by aerobic granular sludge. Biotechnol. Bioeng..

[39] Liu W.T., Nielsen A.T., Wu J.H., Tsai C.S., Matsuo Y., Molin S. (2001). *In situ* identification of polyphosphate- and polyhydroxyalkanoate-accumulating traits for microbial populations in a biological phosphorus removal process. Environ. Microbiol..

[40] Chung J., Kim Y., Lee D., Shim H., Kim J. (2006). Characteristics of Denitrifying Phosphate Accumulating Organisms in an Anaerobic-Intermittently Aerobic Process. Environ. Eng. Sci..

[41] Kim J.M., Lee H.J., Kim S.Y., Song J.J., Park W., Jeon C.O. (2010). Analysis of the fine-scale population structure of “candidatus accumulibacter phosphatis” in enhanced biological phosphorus removal sludge, using fluorescence *in situ* hybridization and flow cytometric sorting. Appl. Environ. Microbiol..

[42] Schroeder S., Ahn J., Seviour R.J. (2008). Ecophysiology of polyphosphate-accumulating organisms and glycogen-accumulating organisms in a continuously aerated enhanced biological phosphorus removal process. J. Appl. Microbiol..

[43] Breus N.A., Ryazanova L.P., Suzina N.E., Kulakovskaya N.V., Valiakhmetov A.Y., Yashin V.A., Sorokin V.V., Kulaev I.S. (2011). Accumulation of inorganic polyphosphates in Saccharomyces cerevisiae under nitrogen deprivation: Stimulation by magnesium ions and peculiarities of localization. Microbiology.

[44] Breus N.A., Ryazanova L.P., Dmitriev V.V., Kulakovskaya T.V., Kulaev I.S. (2012). Accumulation of phosphate and polyphosphate by Cryptococcus humicola and Saccharomyces cerevisiae in the absence of nitrogen. FEMS Yeast Res..

[45] Smith S.A., Morrissey J.H. (2007). Sensitive fluorescence detection of polyphosphate in polyacrylamide gels using 4′,6-diamidino-2-phenylindol. Electrophoresis.

[46] Alvarez S., Jerez C.A. (2004). Copper ions stimulate polyphosphate degradation and phosphate efflux in Acidithiobacillus ferrooxidans. Appl. Environ. Microbiol..

[47] Cosmidis J., Benzerara K., Menguy N., Arning E. (2013). Microscopy evidence of bacterial microfossils in phosphorite crusts of the Peruvian shelf: Implications for phosphogenesis mechanisms. Chem. Geol..

[48] Ge H., Batstone D.J., Keller J. (2015). Biological phosphorus removal from abattoir wastewater at very short sludge ages mediated by novel PAO clade Comamonadaceae. Water Res..

[49] Rivadeneyra A., Gonzalez-Martinez A., Gonzalez-Lopez J., Martin-Ramos D., Martinez-Toledo M.V., Rivadeneyra M.A. (2014). Precipitation of phosphate minerals by microorganisms isolated from a fixed-biofilm reactor used for the treatment of domestic wastewater. Int. J. Environ. Res. Public Health.

[50] Zhang H., Lo V.K., Thompson J.R., Koch F.A., Liao P.H., Mavinic D.S., Atwater J.W. (2015). Recovery of phosphorus from dairy manure: A pilot- scale study. Environ. Technol..

[51] Malley D.F., Lockhart L., Wilkinson P., Hauser B. (2000). Determination of carbon, carbonate, nitrogen, and phosphorus in freshwater sediments by near-infrared reflectance spectroscopy: Rapid analysis and a check on conventional analytical methods. J. Paleolimnol..

[52] Schuster K.C., Urlaub E., Gapes J.R. (2000). Single-cell analysis of bacteria by Raman microscopy: Spectral information on the chemical composition of cells and on the heterogeneity in a culture. J. Microbiol. Methods.

[53] Majed N., Matthäus C., Diem M., Gu A.Z. (2009). Evaluation of intracellular polyphosphate dynamics in enhanced biological phosphorus removal process using Raman microscopy. Environ. Sci. Technol..

[54] Majed N., Gu A.Z. (2010). Application of Raman microscopy for simultaneous and quantitative evaluation of multiple intracellular polymers dynamics functionally relevant to enhanced biological phosphorus removal processes. Environ. Sci. Technol..

[55] Comolli L.R., Kundmann M., Downing K.H. (2006). Characterization of intact subcellular bodies in whole bacteria by cryo-electron tomography and spectroscopic imaging. J. Microsc..

[56] Saito K., Ohtomo R., Kuga-Uetake Y., Aono T., Saito M. (2005). Direct labeling of polyphosphate at the ultrastructural level in Saccharomyces cerevisiae by using the affinity of the polyphosphate binding domain of Escherichia coli exopolyphosphatase. Appl. Environ. Microbiol..

[57] Forbes C.M., O’Leary N.D., Dobson A.D., Marchesi J.R. (2009). The contribution of “omic”-based approaches to the study of enhanced biological phosphorus removal microbiology: Minireview. FEMS Microbiol. Ecol..

[58] McMahon K.D., Dojka M.A., Pace N.R., Jenkins D., Keasling J.D. (2002). Polyphosphate Kinase from Activated Sludge Performing Enhanced Biological Phosphorus Removal. Appl. Environ. Microbiol..

[59] Wilmes P., Wexler M., Bond P.L. (2008). Metaproteomics provides functional insight into activated sludge wastewater treatment. PLoS ONE.

[60] Wexler M., Richardson D.J., Bond P.L. (2009). Radiolabelled proteomics to determine differential functioning of Accumulibacter during the anaerobic and aerobic phases of a bioreactor operating for enhanced biological phosphorus removal. Environ. Microbiol..

